# Investigation on Sub-Solvus Recrystallization Mechanisms in an Advanced γ-γ*’* Nickel-Based Superalloy GH4151

**DOI:** 10.3390/ma13204553

**Published:** 2020-10-14

**Authors:** Shaomin Lv, Jinbin Chen, Xinbo He, Chonglin Jia, Kang Wei, Xuanhui Qu

**Affiliations:** 1 Institute for Advanced Materials and Technology, University of Science and Technology Beijing, Beijing 100083, China; b20170563@xs.ustb.edu.cn (S.L.); quxh@ustb.edu.cn (X.Q.); 2 Science and Technology on Advanced High Temperature Structural Materials Laboratory, Beijing Institute of Aeronautical Materials, Beijing 100094, China; weikang@biam.ac.cn; 3 State Key Laboratory for Advanced Metals and Materials, University of Science and Technology Beijing, Beijing 100083, China; b20170439@xs.ustb.edu.cn

**Keywords:** GH4151 superalloy, dynamic recrystallization, gamma prime precipitates, heteroepitaxial dynamic recrystallization, step-shaped structures

## Abstract

Sub-solvus dynamic recrystallization (DRX) mechanisms in an advanced γ-γ*’* nickel-based superalloy GH4151 were investigated by isothermal compression experiments at 1040 °C with a strain rate of 0.1 s^−1^ and various true strain of 0.1, 0.3, 0.5, and 0.7, respectively. This has not been reported in literature before. The electron backscatter diffraction (EBSD) and transmission electron microscope (TEM) technology were used for the observation of microstructure evolution and the confirmation of DRX mechanisms. The results indicate that a new dynamic recrystallization mechanism occurs during hot deformation of the hot-extruded GH4151 alloy. The nucleation mechanism can be described as such a feature, that is a primary γ*’* (Ni_3_(Al, Ti, Nb)) precipitate embedded in a recrystallized grain existed the same crystallographic orientation, which is defined as heteroepitaxial dynamic recrystallization (HDRX). Meanwhile, the conventional DRX mechanisms, such as the discontinuous dynamic recrystallization (DDRX) characterized by bulging grain boundary and continuous dynamic recrystallization (CDRX) operated through progressive sub-grain merging and rotation, also take place during the hot deformation of the hot-extruded GH4151 alloy. In addition, the step-shaped structures can be observed at grain boundaries, which ensure the low-energy surface state during the DRX process.

## 1. Introduction

Polycrystalline γ-γ’ nickel-based superalloys are widely used for manufacturing critical rotating components of modern aero-engines and gas-turbines due to their excellent mechanical properties and good oxidation resistance at high temperatures [1,2]. However, next generation engines are increasingly demanding, and a higher temperature capacity of turbine disks is required in order to improve the engine performance. Traditionally used alloys, such as Inconel 718 (limited to 650 °C) [3], 718Plus (limited to 710 °C) [4], Udimet 720Li (limited to 730 °C) [5,6], GH4065 [7], and AD730 (limited to 750 °C) [8] are already pushed towards their limits in view of the stability of the microstructure and mechanical properties, resulting in the development of an advanced nickel-based superalloy.

GH4151 alloy, a heavily-alloyed polycrystalline γ-γ’ nickel-based superalloy, is newly developed as a cast and wrought (C&W) superalloy used for turbine disks serviced up to 800 °C [9]. The volume fraction of γ’ phase reaches 55%, near the limit of thermal workability of C&W superalloys. It is acknowledged that the mechanical properties of the critical rotative parts primarily depend on the microstructure developed during thermomechanical processing (TMP). The incoherent primary γ’ particles, pinning at the grain boundaries or triple-junction, known as the Smith–Zener model, can contribute to grain size control and the reduction of deformation resistance during TMP. However, the understanding of DRX mechanisms containing large primary γ’ with diameter ≥ 1.0 μm is quite complex and still not clear.

Generally, it has been widely accepted that discontinuous DRX (DDRX) and continuous DRX (CDRX) are considered as two probably nucleation mechanisms in the DRX process of nickel-based superalloys [10,11]. DDRX involves distinct nucleation and growth stages, which occur in metals with low or medium stacking fault energy [12]. CDRX is deemed as a recovery process and resulted in the generation of high angle grain boundaries (HAGBs) and new grains, which operates through continuous absorption of dislocations in sub-grain boundaries [13]. The effect of hot deformation parameters (deformation temperature, strain and strain rate, etc.) on the DRX of polycrystalline γ-γ’ nickel-based superalloys has been extensively investigated [4,12,14,15,16]. It is concluded that DDRX, characterized by serrated and bulging grain boundaries, is the dominant nucleation mechanism of DRX in nickel-based alloy, while CDRX carried out through progressive sub-grain rotation is only an assistant one [12,17,18]. Meanwhile, the second phase particles (carbides, nitrides and γ’ precipitates) also play significant roles in DRX. Humphreys et al. [19] demonstrated that the existence of a critical value of the transition from accelerated to retarded recrystallization, if *F*_V_/*r* < 0.2 μm^−1^ (*F*_V_ denotes the volume fraction of particles and *r* denotes particle radius), recrystallization can be accelerated by second phase particles and not vice versa. The mechanism of particle stimulated nucleation (PSN), characterized by a misorientation gradient surrounding of large particles, is also widely observed in the previous literature [20,21,22,23]. Meanwhile, recrystallized grains derived from PSN maintain no specific orientation relationship with the involved second phase particles. In addition, Dahlén et al. [24] investigated the effect of γ′ particles dispersions on DRX behavior of the nickel-based superalloy IN 738 LC, and demonstrated that the kinetics of recrystallization are independent of the average size of primary γ′ particles within 0.2–0.6 μm. Lindsley et al. [25] studied sub-solvus recrystallization mechanisms in Udimet720Li, and found that the growth of recrystallized grains occurred primarily by boundary looping of γ’ precipitate leaving the γ’ misoriented with the new matrix. Charpagne et al. [26] observed the primary γ’ particles induced the occurrence of heteroepitaxial recrystallization (HERX) at low strains in René 65, and discovered the same crystallographic orientation between the primary γ’ particles and their host DRX grains. Li et al. [27] investigated the effect of different morphology and sizes of γ’ phase on DRX of FGH4096 superalloy during the sub-solvus temperature range deformation, and concluded that coarse γ’ particles can promote the occurrence of DRX behavior. Lv et al. [28] observed that the occurrence of γ’ particle-induced DRX (PIDRX) at the sub-solvus temperature of GH4065 alloy, deriving from the formation of sub-grains accelerated by γ’, precipitates pinning dislocations.

Although several researchers have already investigated the mechanisms of DRX nucleation in nickel-based superalloys, it is unfortunate that the understanding is insufficient on DRX mechanisms of GH4151 alloy containing large number of primary γ’ particles, γ + γ’ dual-phase microstructure. Therefore, further systematic investigations on the microstructural evolution and nucleation mechanisms of GH4151 alloy during isothermal compression are extremely necessary. The aim of the present study is to illuminate the DRX nucleation mechanisms of GH4151 alloy, and focus on the contribution of large γ’ particles with the diameter of 1–3 μm by thermal simulation experimentation at sub-solvus temperatures. The electron backscatter diffraction (EBSD) and transmission electron microscope (TEM) were used for the observation of microstructure evolution and the confirmation of DRX mechanisms.

## 2. Materials and Experimental Procedure

### 2.1. Material

**Figure 1:**
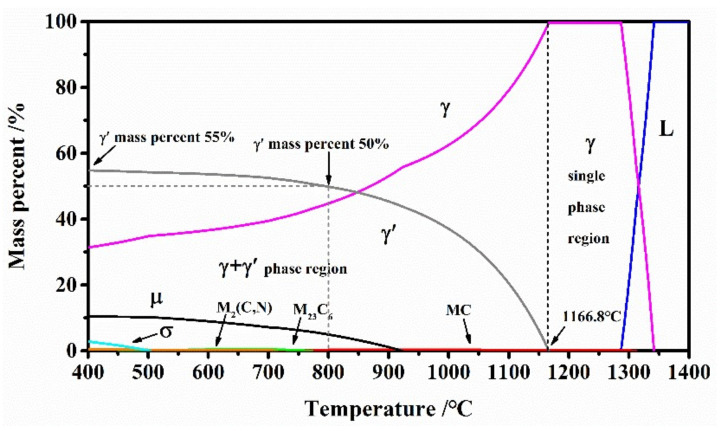
Phase transition diagram of GH4151 alloy plotted by JMatPro software.

**Figure 2:**
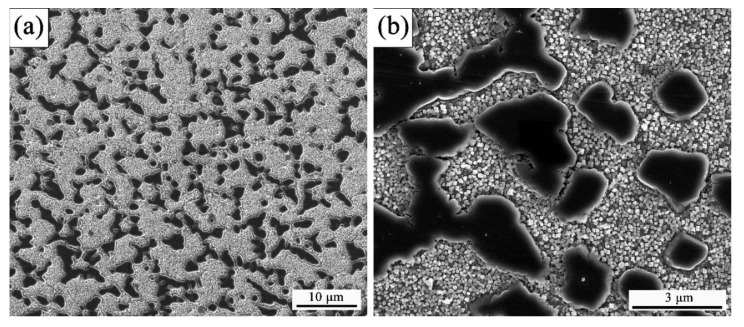
The as-received microstructure of the hot-extruded GH4151 alloy at different magnifications: (**a**) 5000×; (**b**) 25000×.

GH4151 alloy is a novel polycrystalline γ-γ’ nickel-based superalloy, of which chemical composition (wt.%) used in this study is listed in Table 1. The thermodynamic equilibrium phase diagram predicts the phase composition of the GH4151 alloy versus temperature shown in Figure 1, which is plotted by JMatPro software (version 7.0, Sente Software Ltd., Guildford, U.K.) according to Table 1. As illustrated in Figure 1, it can be determined that the volume fraction of γ’ phase reaches 55% and the γ’ solvus temperature is about 1166.8 °C. The vacuum induction melting (VIM), vacuum arc remelting (VAR) and diffusion homogenization were used for preparing the as-received GH4151 alloy (BIAM, Beijing, China). The VIM + VAR ingot is then converted into a billet with a nominal diameter of 60 mm. The as-received microstructure of the hot-extruded GH4151 alloy is shown in Figure 2, consisting of fine grains (ranged from 4 to 8 μm) and primary γ’ particles (1–3 μm) pinned at the grain boundaries or triple-junctions. Cylindrical compression specimens of 8 mm in diameter and 12 mm in height are machined from the as-received hot-extruded billet.

### 2.2. Hot Compression

**Figure 3:**
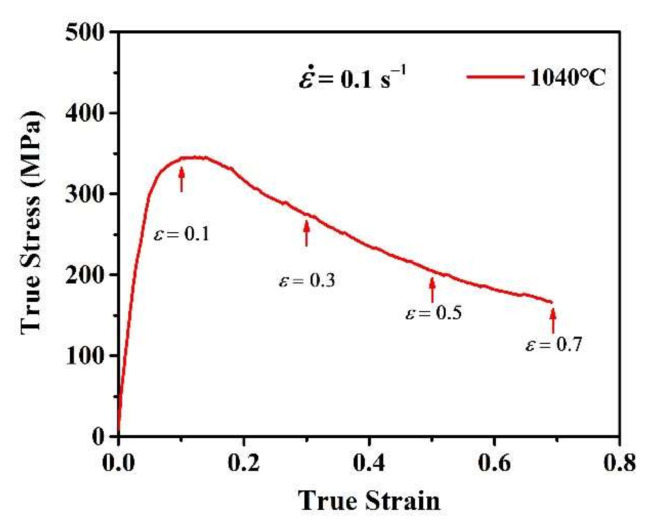
The true stress-strain curves of the hot-extruded GH4151 alloy in this work.

The isothermal compression tests were carried out on a Gleeble-3800 thermomechanical simulator (DSI, St. Paul, Minnesota, USA) at a strain rate of 0.1 s^−1^ and temperature of 1040 °C, with various true strain of 0.1, 0.3, 0.5 and 0.7, respectively. The true stress–strain curves are shown in Figure 3. In order to minimize the friction effect during isothermal compression, high quality graphite foils were used as a lubricant between the specimen and anvils. Cylindrical compression specimens were heated up to the experimental temperature with a heating rate of 10 °C/s and held for 300 s to eliminate the temperature gradient before deformation. After isothermal compression, all the specimens were immediately water-quenched within 3 s to freeze the microstructure.

### 2.3. Microstructure Observation

The hot deformed specimens were cut along longitudinal direction through the center to prepare specimens for electron backscattered diffraction (EBSD) and transmission electron microscopy (TEM). Specimens for EBSD were achieved by mirror polishing followed by electrochemical polishing in 20% H_2_SO_4_ and 80% CH_3_OH at 10–20 V for 10–15 s. EBSD studies are performed on a FEI Quanta 650 FEG-SEM scanning electron microscope (FEI Corporation, Hillsboro, OR, USA) equipped with a HKL Channel 5 software system, and the step size is 0.2 μm. Subsequently, the collected data were processed and clean up with TSL OIM Analysis software (6.2.0 x86 version, EDAX Inc., Draper, UT, USA). Grain CI standardization was operated and then grain dilation was carried out in post process of the cleanup of the OIM images. The grain tolerance angle and minimum grain size are set to 5 and 2, respectively. TEM foils were prepared by grinding to a thickness of 50 μm and then ion milling. TEM observations were performed with a Talos F200x G2 transmission electron microscope (Thermo Fisher, Waltham, MA, USA) operated at 200 kV.

## 3. Results and Discussion

### 3.1. Microstructure Evolution

**Figure 4:**
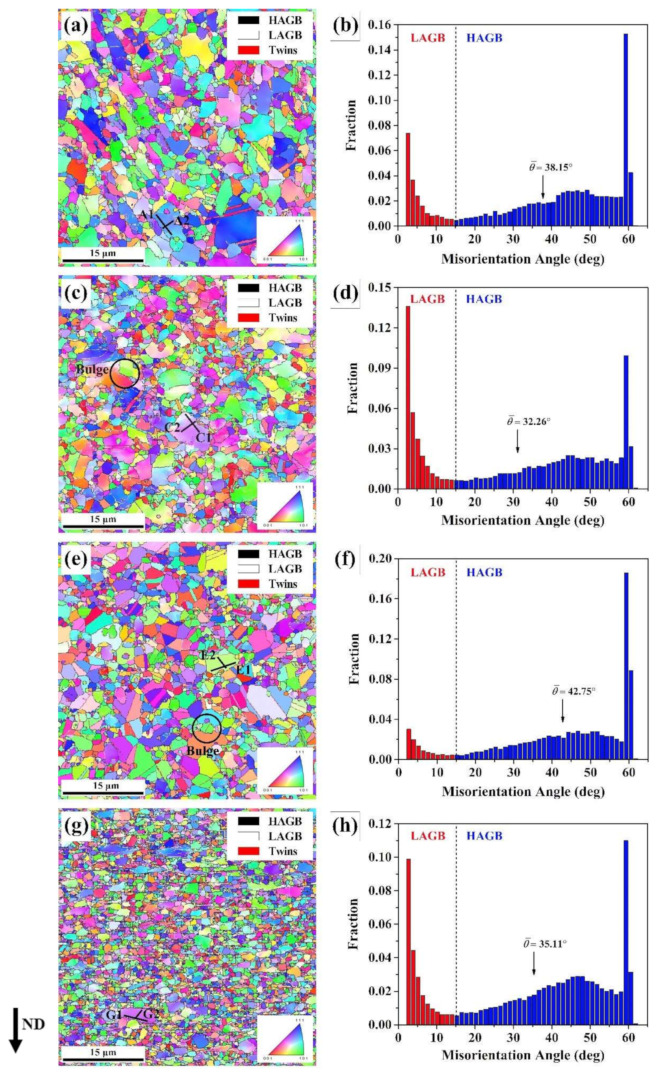
OIM (Orientation Imaging Microscopy) maps and the distribution of misorientation angle of GH4151 alloy after deformation at 1040 °C, 0.1 s^−1^ and different strain of (**a**,**b**) 0.1; (**c**,**d**) 0.3; (**e**,**f**) 0.5; (**g**,**h**) 0.7.

In order to study the microstructural evolution of dynamic recrystallization (DRX) at different true strains, the isothermal compression tests with various true strain of 0.1, 0.3, 0.5 and 0.7 were carried out, respectively. Figure 4 illustrates the orientation imaging microscopy (OIM) maps and misorientation angle distribution of homogenized alloy deformed to different true strains ranging from 0.1 to 0.7 at 1040 °C with the strain rate of 0.1 s^−1^, respectively. In this study, the reference direction for colors in Figure 4 is the compression axis, and the ND direction indicates the direction along compression axis.

At the true strain of 0.1, as shown in Figure 4a, it can be discovered that the original grains are elongated and the deformed grains are surrounded by refined DRX grains. This can be described as “necklace structure” [29], illuminating the occurrence of typical DDRX mechanism. Meanwhile, the low angle grain boundaries (LAGBs, <10°, plotted by thin-white boundaries) can be observed in deformed grains interior. As shown in Figure 4c, when the true strain increased to 0.3, the density of LAGBs is relatively high, which indicates the formation of dislocation substructures and sub-grain boundaries in deformed grains. This is due to the deformation storage energy increased with the true strain increasing to 0.3, resulting in the strong driving force for the movements of dislocation and the migration of grain boundary [30]. With the true strain of 0.5, the fraction of DRX grains increases and a number of DRX grains grow up, as shown in Figure 4e. However, when the true strain further increases to 0.7, a large number of DRX grains are refined, as depicted in Figure 4g. Meanwhile, the relatively high density of LAGBs can be observed in deformed grains interior, which indicates the occurrence of the next round of DRX process. Furthermore, Figure 4b,d,f,h depict the grain misorientation angle distributions of the specimens with the true strain of 0.1, 0.3, 0.5, and 0.7, respectively. The average misorientation angles at 0.1, 0.3, 0.5, and 0.7 can be identified as 38.15°, 32.26°, 42.75°, and 35.11°, respectively. Obviously, the average misorientation angle dose not vary monotonically as the true strain increasing. The evolution characteristic of the average misorientation angle is considered to be associated with the DRX grain growth and volume fraction [31]. This demonstrates the occurrence of the next round of the DRX process when the true strain increases to 0.7.

**Figure 5:**
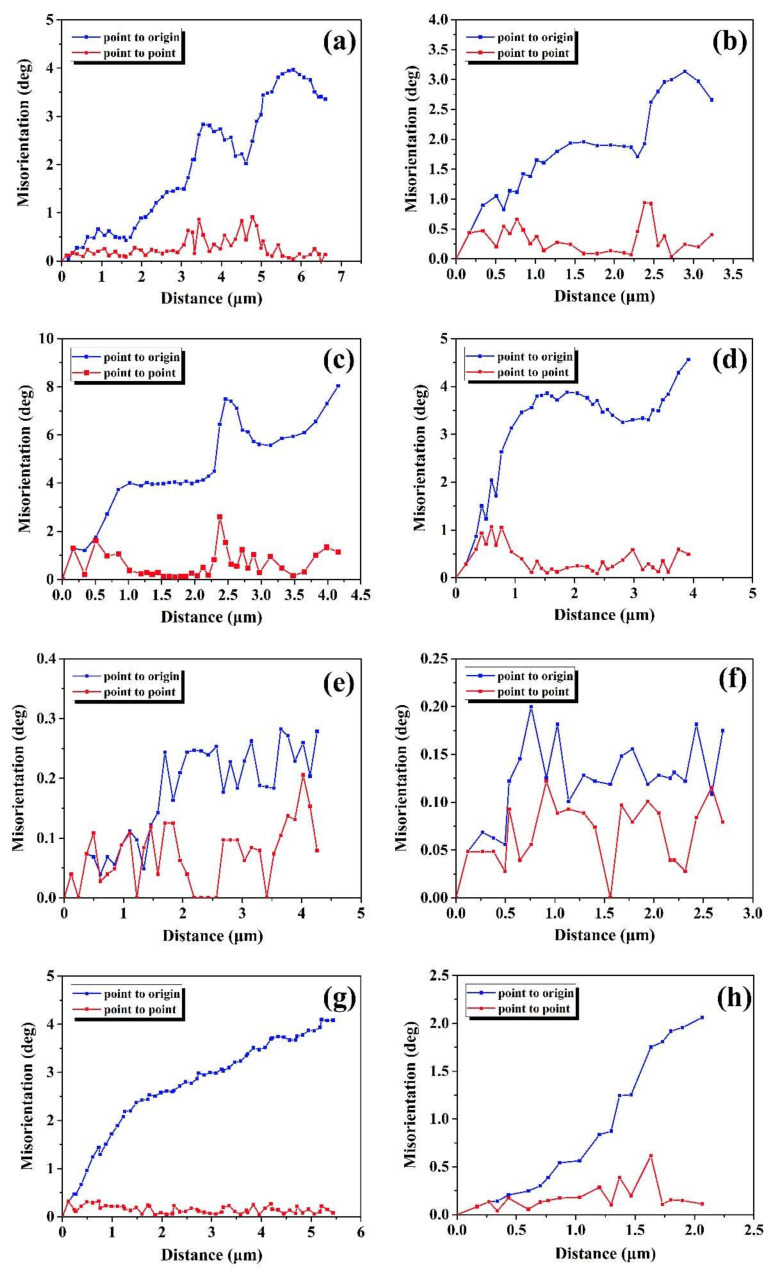
Evolution of misorientation corresponding to the lines indicated in Figure 4, severally: (**a**) A1; (**b**) A2; (**c**) C1; (**d**) C2; (**e**) E1; (**f**) E2; (**g**) G1; (**h**) G2.

It is widely acknowledged that the CDRX nucleation mechanism is related with the evolution of sub-grains, which can be estimated by misorientation analysis [32,33,34]. Figure 5 depicted that the point to point misorientations and the point to origin misorientations corresponding to the lines indicated in Figure 4. The point to origin misorientation of a length of 6 μm exceeds 4° along the grain boundaries and that exceeds 3° in grain interior, as plotted in Figure 5a,b. Meanwhile, it is difficult for the point to point misorientations to exceed 1° whether along the line A1 or line A2. This reveals that the sub-grain rotation is not obvious at the true strains of 0.1. When the true strain increases to 0.3, it can be found that the point to origin misorientations increase obviously, as illustrated in Figure 5c, demonstrating that misorientation accumulates with the occurrence of progressive sub-grain merging and rotation. This means the occurrence of CDRX operated via progressive sub-grain merging and rotation [35], and further indicates that the sub-structures and the DRX nuclei growth process may occur by means of accumulating adequate dislocations to transfer to HAGBs [12]. Thus, it can be concluded that the progressive sub-grain merging and rotation becomes stronger with the true strain increasing from 0.1 to 0.3. In addition, the misorientation jumps can be obviously observed at the distance range of (2.25–2.5) μm along the grain boundaries in Figure 5c. The similar evolution characteristic of the misorientation jumps is also shown in Figure 5a,b. This phenomenon indicates that different orientation bands exist in the deformed grains, resulting from different slip systems activated for coordinating the strain between the adjacent grains [28,36].With the true strain of 0.5, it is easily observed that the point to origin misorientations and the point to point misorientations obviously decreases, and scarcely exceed 0.3° both along the E1 line and E2 line in Figure 4e, as shown in Figure 5e,f. It suggested that the sub-grain merging and rotation becomes slight at the true strain of 0.5. This phenomenon demonstrates that the deformed grains transform into the dislocation-free DRX grains [28]. With the true strain further increasing from 0.5 to 0.7, it can be found that the point to origin misorientations obviously increases, and the point to point misorientations scarcely exceed 0.5 both along the G1 line and G2 line in Figure 4g, as shown in Figure 5g,h. This implies that the existence of a sub-structure at grain interior and the progressive sub-grain merging and rotation become stronger again, further indicating that the next DRX process begins by stored deformation energy.

**Figure 6:**
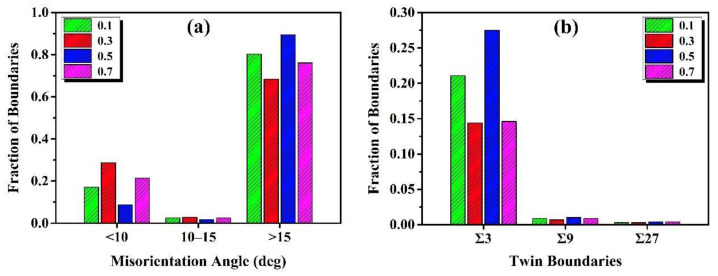
The distribution of grain boundary misorientation (**a**) and the fraction of Σ3, Σ9 and Σ27 boundaries (**b**) of GH4151 samples deformed at different strains of 0.1, 0.3, 0.5 and 0.7, respectively.

In order to further analyze the grain boundary characteristics involved in DDRX and CDRX mechanisms, the distribution of grain boundary misorientation is illustrated in Figure 6a. It can be easily found that the fraction of LAGBs increase to 28.7% at the true strain from 0.1 to 0.3, which results from the accumulation of dislocations leading to the generation of LAGBs. Meanwhile, the fraction of medium angle grain boundaries (MAGBs, 10°~15°) reaches the maximum value of 2.9% at the true strain of 0.3, indicating that the CDRX mechanism carried out through sub-grain rotation becomes stronger, resulting in the generation of sub-grain structures. Nevertheless, when the true strain increasing to 0.5, the fraction of LAGBs decreases sharply to 8.8% and that of HAGBs increase to 89.4%, which is related to the generation of dislocation-free DRX grains due to LAGBs transforming to HAGBs. With the true strain further increasing from 0.5 to 0.7, it is noteworthy that the fraction of LAGBs increases again to 21.4% and that of MAGBs increases to 2.5%. This mainly results from the generation of dislocations in the substructure and sub-grains in the grain interior, once again when the true strain increases to 0.7. This is to say, the next DRX process begins at the true strain of 0.7. It is well known that the MAGBs can be considered as a signal of the CDRX mechanism. Thus, from the above analysis, the CDRX mechanism take place easily at the initial stage of DRX process, such as at the true strain of 0.1, 0.3, and 0.7. However, the fraction of MAGBs is always less than 3.0%, as illustrated in Figure 6a. Thus, it can be concluded that the CDRX mechanism operated by progressive sub-grain rotation only plays an assistant role at all stages of deformation.

The fraction of Σ3, Σ9, and Σ27 boundaries at true strains of 0.1, 0.3, 0.5, and 0.7 are shown in Figure 6b. It can be observed that the fraction of Σ3 boundaries decrease from 21.1% at the true strain of 0.1 to 14.4% at the true strain of 0.3. This is due to the loss of an Σ3 twin relationship of original twin boundaries resulting from the rotation of grains [12]. Nevertheless, the fraction of Σ3 boundaries reaches the maximum value of 27.5%, which is related to the nucleation and growth of new twins during the growth process of DRX grains. When the true strain increases to 0.7, the fraction of Σ3 boundaries decreases to 14.6%, which is quite similar to that at the true strain of 0.3. Furthermore, the fractions of Σ9 boundaries and Σ27 are less than 1.0% at all stages of deformation, as shown in Figure 6a. This indicates that the interactions between Σ3*^n^* boundaries are weaker. Therefore, it is concluded that the generation of Σ3 boundaries is attributed to the ‘growth accident’ mechanism [28].

### 3.2. DRX Nucleation Mechanisms

It has been widely accepted that the DDRX and CDRX mechanisms are considered as the main DRX mechanisms in nickel-based superalloys [11,33]. Meanwhile, the role of γ’ precipitates in DRX process has been investigated in previous studies [26,27,37,38]. It is a “dual-phase” microstructure characterized by the fine grains (ranged from 4 to 8 μm) with large of primary γ’ particles (with 1 to 3 μm) of the hot-extruded GH4151 alloy in present study. For the purpose of further confirming the sub-solvus DRX mechanisms of the hot-extruded GH4151 alloy during hot deformation, TEM observations are carried out.

#### 3.2.1. The DRX Mechanisms at True Strain of 0.1

**Figure 7:**
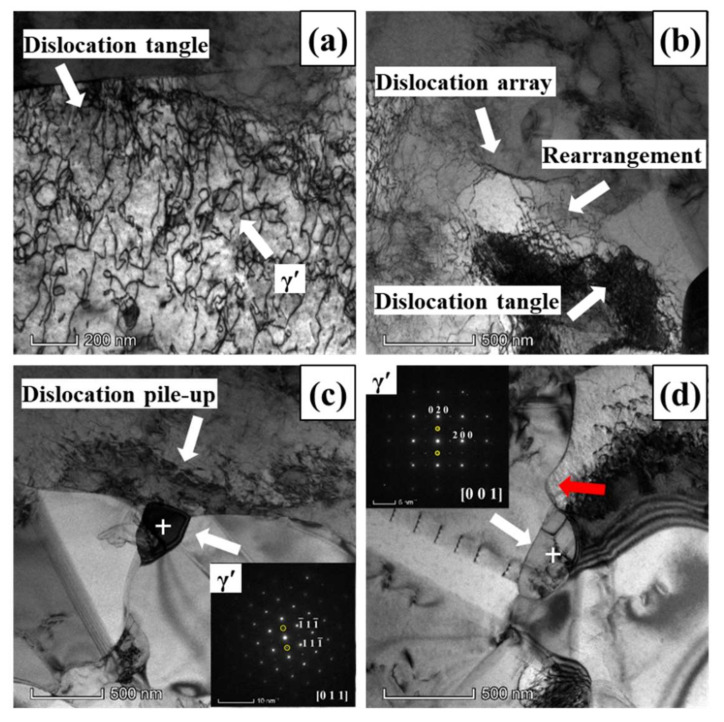
TEM morphology of GH4151 alloy after deformation at 1040 °C, 0.1 s^−1^ and strain of 0.1: (**a**) Dislocation tangle; (**b**) Dislocations; (**c**) Dislocation pile-up; (**d**) γ’ particle pinning the grain boundary.

Figure 7 illustrated the TEM images and selected area electron diffraction (SAED) patterns of GH4151 alloy after hot deformation at the true strain of 0.1. As depicted in Figure 7a, it can be observed that high density dislocations near the origin straight grain boundary and the interaction between fine γ’ particle and dislocations at the grain interior. Figure 7b shows the dislocations evolution at the grain interior, which can be characterized as: dislocations accumulated→dislocation tangled→dislocations rearranged→the formation of dislocations array. The dislocations tangle and pile-up and γ’ particle (indicated by the SAEDs in Figure 7c) pinning at the triple junction. The dislocations evolution exhibits work hardening (WH) at the initial stage of hot deformation, and dynamic recovery (DRV) operated by dislocations rearrangement and annihilation in order to reduce the remnant stored energy [19]. In addition, it is easily observed that the migration of grain boundary was hindered by γ’ particle (indicated by the SAEDs in Figure 7d), and the grain boundary sends dislocations into the grain interior to relax the stress field caused by γ’ particle pinning the grain boundary.

#### 3.2.2. The DRX Mechanisms at True Strain of 0.3

**Figure 8:**
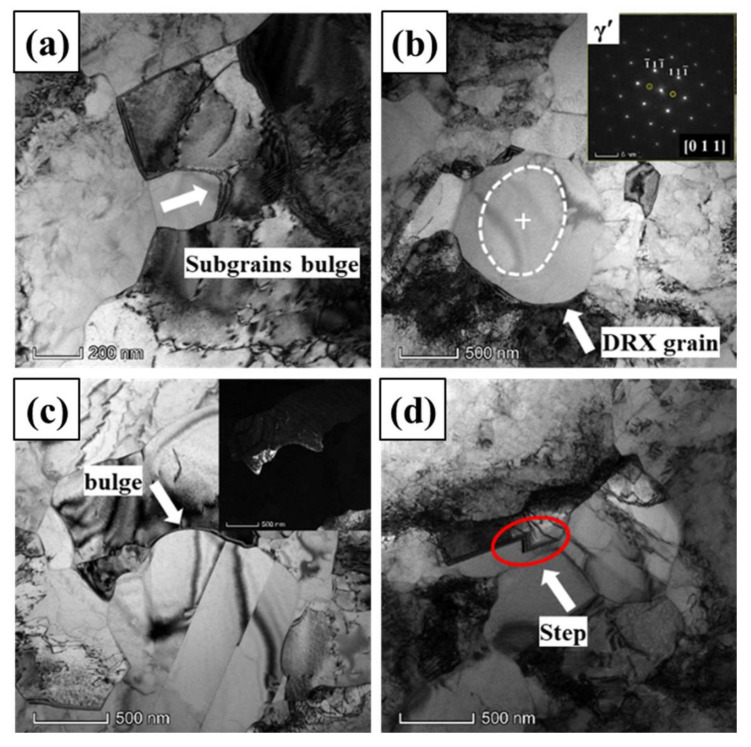
TEM images of GH4151 alloy after deformation at 1040 °C, 0.1 s^−1^ and strain of 0.3: (**a**) Subgrain bulge; (**b**) DRX grain; (**c**) Bulge; (**d**) Step-shaped grain boundary.

When the true strain increases to 0.3, the typical TEM images and the SAEDs of GH4151 alloy after hot deformation at 1040 °C, 0.1 s^−1^ are shown in Figure 8. As shown in Figure 8a, sub-grains formed, and a mass of dislocations accumulated and rearranged in the grain interior. The sub-grain boundary can transform into HAGBs operating by consuming dislocations, as shown in Figure 8a. The dislocations evolution indicated that the nucleation mechanism of CDRX plays a significate role in the DRX process at true strain of 0.3. In addition, the bulging grain boundary can be observed as illustrated in Figure 8a,c, which is the typical characteristic of the DDRX mechanism. Meanwhile, the part of bulging grain boundary was separated by twin, as shown in Figure 8c. This approved that a twin can facilitate the DRX process by accelerating the bulging and the bulging part separated from the original grain [39]. It is particularly observed that the dislocations-free DRX grain hosted by γ*’* phase (indicated by the SAEDs in Figure 8b), and fine tertiary γ*’* precipitates as shown in the white circle outside. In addition, it is noteworthy that the formation of the step-shaped grain boundary can be found in Figure 8d.

#### 3.2.3. The DRX Mechanisms at True Strain of 0.5

**Figure 9:**
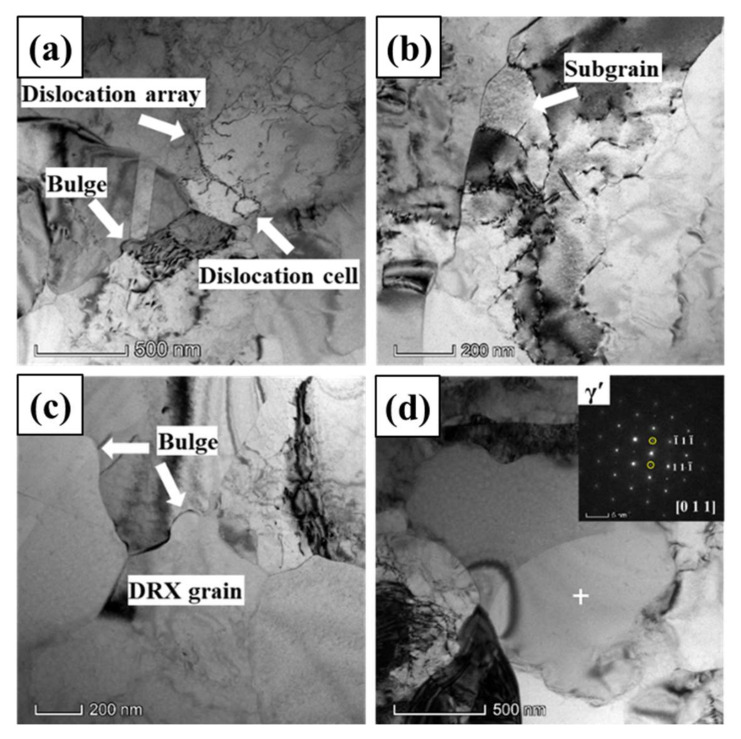
TEM morphology of GH4151 alloy after deformation at 1040 °C, 0.1 s^−1^ and strain of 0.5: (**a**) Bulge and dislocations; (**b**) Subgrain; (**c**) Bulge and DRX grain; (**d**) DRX grain.

Figure 9 exhibits the typical TEM micrographs and the SAED patterns of GH4151 alloy after hot deformation at the true strain of 0.5. As illustrated in Figure 9a, the dislocations evolution can be observed, which is described as: random dislocation→dislocations network and array→dislocation cell. Meanwhile, the bulging grain boundary associating with twin can also be found, as indicated in Figure 9a, which consisting with twin can promote the process of DRX by accelerating the bulging [39]. As shown in Figure 9b, the sub-grain boundary transforms from the rearranged dislocations, and then converts into HAGBs through absorbing lattice dislocations [33]. It indicates that the CDRX nucleation mechanism takes place at hot deformation at 1040 °C, 0.1 s^−1^ and strain of 0.5. Moreover, the appearance of bulging grain boundary can be found in Figure 9c, which demonstrated that the DDRX nucleation mechanism takes place, as well as the nucleation by CDRX. In addition, it is particularly observed that the dislocation-free DRX grain contains two areas: (i) γ*’* phase; (ii) γ phase containing a lot of fine tertiary γ*’* precipitates. The SAED pattern indicates that the two areas maintain the same crystallographic orientation, as shown in Figure 9d, and the similar structure has already been observed in Figure 8b.

#### 3.2.4. The DRX Mechanisms at True Strain of 0.7

**Figure 10:**
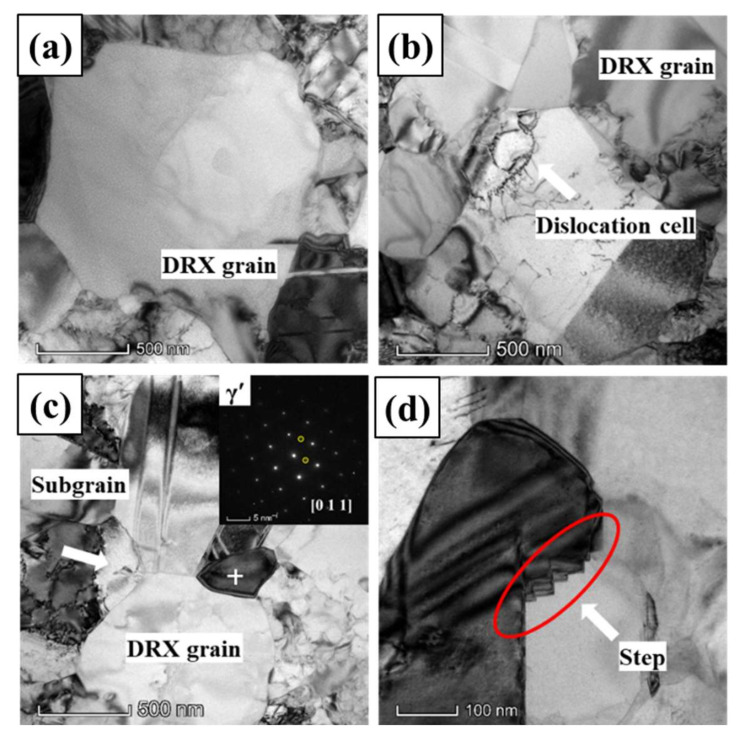
TEM images of GH4151 alloy after deformation at 1040 °C, 0.1 s^−1^ and strain of 0.7: (**a**) DRX grain; (**b**) Dislocation cell and DRX grain; (**c**) subgrain and DRX grain; (**d**) Step-shaped grain boundary.

When the true strain increasing to 0.7, Figure 10 shows that TEM images and the SAED pattern of GH4151 alloy after hot deformation at the true strain of 0.7. As depicted in Figure 10a, it can be observed that the generation of sub-grain and the γ*’* precipitate (indicated by the SAEDs in Figure 10c) pinned at triple-junction. It is interesting to note that the dislocation-free DRX grain hosted by γ*’* precipitate can be found in Figure 10a, and the similar structure has already been observed in Figure 8b and Figure 9d. Thus, it can be concluded that the new DRX mechanism plays an important role during the DRX process of the hot-extruded GH4151 alloy. Based on the discussions in Section 3.2.2 and Section 3.2.3, this new DRX mechanism can be defined as heteroepitaxial dynamic recrystallization (HDRX), which is characterized by a primary γ’ (Ni_3_(Al, Ti, Nb)) particle embedded in a recrystallized grain and existing the same crystallographic orientation.

In addition, the step-shaped structure can also be observed in Figure 10d, and the similar structure has already been observed in Figure 8d. The formation of step-shaped grain boundary resulted from ensuring more interface to be low energy close-packed surface, in order to reduce the energy of grain boundary interface [40]. The formation of step-shaped grain boundary can be observed in hot deformation process, as depicted in Figure 8d and Figure 10d. Based on the theory of coincidence-site lattice (CSL), the grain boundary would be inclined to generate along the closely packed plane to contain the maximum coincident lattice atoms, in order to reduce the grain boundary energy [40]. Obviously, the grain boundary energy is closely related to the amount of coincident lattice atoms at grain boundary. In other words, the less distortion degree of the atomic arrangement, the lower the grain boundary energy. However, when the grain boundary orientation deviates from the closely packed plane, the step-shaped structure can be generated at grain boundary to decrease the grain boundary energy. As shown in Figure 8d, the step-shaped grain boundaries form via absorbing dislocations. Wei et al. [40] points out the grain boundary dislocation is characterized by specific Burgers vector (*b* = [120]/5), which facilitates the formation of step-shaped grain boundary. Similarly, Qu et al. [14] observed the generation of step-shaped grain boundary resulted from the dislocation interactions in GH4730 alloy.

### 3.3. The Characteristics of HDRX

**Figure 11:**
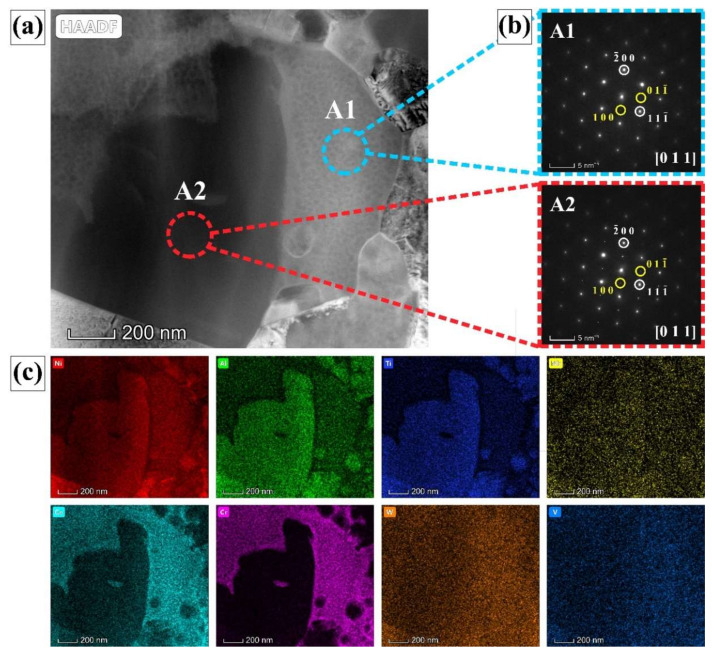
(**a**) HAADF of the dislocation-free DRX grain; (**b**) SAED in [011] zone axis corresponding to the γ matrix (A1) and γ’ precipitate (A2); (**c**) EDS maps of the dislocation-free DRX grain.

**Figure 12:**
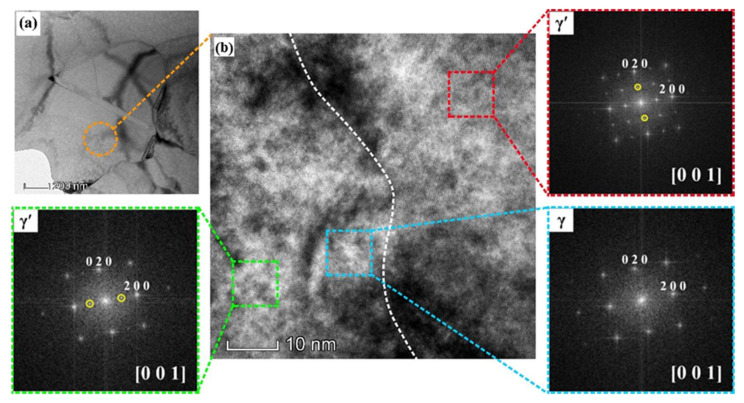
High-resolution TEM at the interface of coherent γ and γ’ phase with corresponding diffraction patterns simulated by fast Fourier transformations: (**a**) HRTEM of the DRX grain; (**b**) HRTEM of the interface corresponding.

To elucidate the characteristics of HDRX mechanism as shown in Figure 8b, Figure 9d and Figure 10a, further investigation can be operated by TEM observation, as shown in Figure 11 and Figure 12. The high angle annular dark field (HAADF) of the dislocation-free DRX grain hosted by γ*’* precipitate is shown in Figure 11a. The SAED pattern indicates corresponding to the matrix is a typical FCC crystal structure within a [011] zone axis, with characteristic superlattice diffraction spot of the γ*’* precipitate, as shown in Figure 11b. The EDS elemental maps of Figure 11c exhibit the formation elements of γ*’* precipitate, such as Al, Ti, and Ni, concentrated in the area of the white circle inside, which indicates this is γ*’* precipitate. Moreover, the area of white circle outside contains large number of fine tertiary γ*’* precipitates, where there concentrate solution strengthening elements, such as Co and Cr, corresponding to the γ matrix. The SAED pattern further confirms the area is the γ matrix, as shown in Figure 11b.

The high-resolution TEM in bright field at the interface of γ’ and γ structure is illustrated in Figure 12. As shown in Figure 12a, a thin twin (indicated as red arrow) can be found crossing the precipitate. Figure 12b illustrates the high-resolution TEM image at the interphase corresponding to the area as indicated in Figure 12a. The FFT (fast Fourier transforms) of three areas indicate the area A, B, and C corresponding to fine tertiary γ’ precipitate, γ matrix, and γ’ precipitate, respectively. Meanwhile, it is particularly noted that the three areas are the typical face centered cubic (FCC) crystal structure within a [001] zone axis, which confirms that the three areas maintain the same crystallographic orientation relationship. Thus, it is proved that the large γ’ precipitate and its hosting DRX grain retain the coherent relationship in crystallography at the γ/γ′ interface. The coherent γ/γ′ interfaces have very low energies [41,42].

In fact, Charpagne et al. observed the primary γ’ particles induced the occurrence of heteroepitaxial recrystallization (HERX) at low strains in René 65. Here, the difference with previous works is been stated as follows. From the view of the initial microstructure, the as-received samples were annealed before the hot isothermal compression. The primary γ’ particles have a spheroidal shape and are all located on grain boundaries or at triple junctions. Charpagne et al. demonstrated that HERX always starts from annealing process and low strain of hot deformation, and then eventually completely disappear at high strains.

However, in our study, the isothermal compression specimens of the hot-extruded GH4151 alloy were not annealed before the hot deformation. The as-received microstructure of the hot-extruded GH4151 alloy consists of fine grains (4–8 μm) and primary γ*’* particles (1–3 μm) pinned at the grain boundaries or triple-junction. Meanwhile, HERX was not found during the hot deformation at low strain of 0.1, but observed at the higher strain of 0.3, 0.5, and 0.7. This indicates that the HDRX mechanism become activated at higher strain. In addition, in this study, the nucleation mechanism can be described as such a feature, that is a primary γ’ (Ni_3_(Al, Ti, Nb)) precipitate embedded in a recrystallized grain existed the same crystallographic orientation. It is demonstrated with the SAED and EDS maps of the dislocation-free DRX grain in this study.

Here, based on the discussions above, it is concluded that multiple mechanisms exist in the deformation process of the hot-extruded GH4151 alloy. The traditional nucleation mechanisms of DDRX and CDRX are observed by EBSD and TEM analysis. The CDRX mechanism carried out through sub-grain merging and rotation takes place easily at the initial stage of DRX process, such as at the true strain of 0.1, 0.3 and 0.7. DDRX, characterized by bulging grain boundary, plays a dominant role in the DRX process, while CDRX is only deemed as an assistant nucleation mechanism. Meanwhile, it is especially noteworthy that a new DRX mechanism, heteroepitaxial dynamic recrystallization (HDRX), takes place simultaneously under the current deformation conditions. In addition, the formation of the step-shaped grain boundary can be found in the DRX process of the hot-extruded GH4151 alloy.

## 4. Conclusions

In order to elucidate the DRX mechanisms of polycrystalline γ-γ’ nickel-based superalloy containing a large number of primary γ’ particles, a novel hot-extruded GH4151 alloy was investigated by isothermal compression tests. This research focuses on the microstructural evolution associated with DRX and the contribution of large γ’ particles at sub-solvus temperatures. The main conclusions are as follows:

- With the true strain increasing, the fraction of LAGBs increases firstly and then decreases, and then rises again, while the fraction of HAGBs evolves inversely. The fraction of MAGBs scarcely exceeds 3.0%, revealing that the CDRX mechanism is an assistant nucleation mechanism of DRX at all stage of deformation.
- The CDRX mechanism operated by progressive sub-grain merging and rotation takes place easily at the initial stage of the DRX process. DDRX, characterized by a bulging grain boundary, plays a dominant role in the DRX process, while CDRX is only deemed as an assistant nucleation mechanism.
- The heteroepitaxial dynamic recrystallization (HDRX) takes place in the deformation process of the hot-extruded GH4151 alloy, which is characterized by a primary γ*’* (Ni_3_(Al, Ti, Nb)) precipitate embedded in a recrystallized grain and having the same crystallographic orientation.
- The step-shaped grain boundary can be observed during the DRX process, which resulted from ensuring more of the interface to be a low-energy, closely packed surface, in order to reduce the energy of the grain boundary interface.

## Figures and Tables

**Table 1:** Chemical composition of the GH4151 superalloy (major elements).

Elements	Ni	C	Cr	Co	Mo	W	Al	Ti	Nb	V
**wt.%**	Bal.	0.07	10.0	15.0	4.5	3.0	3.6	2.8	3.4	0.5
